# Explaining variation in adult *Anopheles* indoor resting abundance: the relative effects of larval habitat proximity and insecticide-treated bed net use

**DOI:** 10.1186/s12936-017-1938-1

**Published:** 2017-07-17

**Authors:** Robert S. McCann, Joseph P. Messina, David W. MacFarlane, M. Nabie Bayoh, John E. Gimnig, Emanuele Giorgi, Edward D. Walker

**Affiliations:** 1 Department of Entomology, Michigan State University, East Lansing, MI USA; 2 Department of Geography, Environment, and Spatial Sciences, Michigan State University, East Lansing, MI USA; 3 Department of Forestry, Michigan State University, East Lansing, MI USA; 4 Centre for Global Health Research, Kenya Medical Research Institute/Centers for Disease Control and Prevention, Kisumu, Kenya; 5 Division of Parasitic Diseases and Malaria, Centers for Disease Control and Prevention, Atlanta, GA USA; 6 Lancaster Medical School, Lancaster University, Lancaster, UK; 7 Department of Microbiology and Molecular Genetics, Michigan State University, 567 Wilson Road, 2215 Biomedical Physical Sciences Building, East Lansing, MI 48824-4320 USA

**Keywords:** Spatial heterogeneity, Larval habitats, Malaria vectors, *Anopheles gambiae*, *Anopheles arabiensis*, *Anopheles funestus*, Generalized linear models, Geostatistical models

## Abstract

**Background:**

Spatial determinants of malaria risk within communities are associated with heterogeneity of exposure to vector mosquitoes. The abundance of adult malaria vectors inside people’s houses, where most transmission takes place, should be associated with several factors: proximity of houses to larval habitats, structural characteristics of houses, indoor use of vector control tools containing insecticides, and human behavioural and environmental factors in and near houses. While most previous studies have assessed the association of larval habitat proximity in landscapes with relatively low densities of larval habitats, in this study these relationships were analysed in a region of rural, lowland western Kenya with high larval habitat density.

**Methods:**

525 houses were sampled for indoor-resting mosquitoes across an 8 by 8 km study area using the pyrethrum spray catch method. A predictive model of larval habitat location in this landscape, previously verified, provided derivations of indices of larval habitat proximity to houses. Using geostatistical regression models, the association of larval habitat proximity, long-lasting insecticidal nets (LLIN) use, house structural characteristics (wall type, roof type), and peridomestic variables (cooking in the house, cattle near the house, number of people sleeping in the house) with mosquito abundance in houses was quantified.

**Results:**

Vector abundance was low (mean, 1.1 adult *Anopheles* per house). Proximity of larval habitats was a strong predictor of *Anopheles* abundance. Houses without an LLIN had more female *Anopheles gambiae* s.s., *Anopheles arabiensis* and *Anopheles funestus* than houses where some people used an LLIN (rate ratios, 95% CI 0.87, 0.85–0.89; 0.84, 0.82–0.86; 0.38, 0.37–0.40) and houses where everyone used an LLIN (RR, 95% CI 0.49, 0.48–0.50; 0.39, 0.39–0.40; 0.60, 0.58–0.61). Cooking in the house also reduced *Anopheles* abundance across all species. The number of people sleeping in the house, presence of cattle near the house, and house structure modulated *Anopheles* abundance, but the effect varied with *Anopheles* species and sex.

**Conclusions:**

Variation in the abundance of indoor-resting *Anopheles* in rural houses of western Kenya varies with clearly identifiable factors. Results suggest that LLIN use continues to function in reducing vector abundance, and that larval source management in this region could lead to further reductions in malaria risk by reducing the amount of an obligatory resource for mosquitoes near people’s homes.

**Electronic supplementary material:**

The online version of this article (doi:10.1186/s12936-017-1938-1) contains supplementary material, which is available to authorized users.

## Background

Malaria prevalence within communities is spatially heterogeneous over a wide range of ecological and epidemiological settings, especially when community-wide transmission intensity is low to moderate [1]. While the recognition of spatial variation in malaria risk predates even the etiology of the disease [2], a number of factors contribute to this spatial heterogeneity. Within communities, socioeconomic and immunological differences affect the prevalence of malaria in people [3–5]. Additionally, the ecologies of the local malaria vectors determine the spatial distribution of malaria, often through the relative juxtaposition of the vectors’ larval habitats and people’s homes [6–9]. Depending on the epidemiological context, closer proximity to larval habitats may lead to increased [10, 11] or decreased [12] incidence of clinical malaria. In either case, it is clear that the spatial distribution of malaria vectors has important implications for malaria transmission.

The spatial distributions of adult malaria vectors are determined, in part, by landform variations through effects on hydrology and the locations of aquatic habitats for *Anopheles* larvae [13]. In landscapes where larval habitats are restricted to a linear feature such as along a river or swamp edge, adult *Anopheles* abundance per house increases with decreasing distance to the river or swamp [6, 14, 15]. A similar relationship exists for some *Anopheles* species in landscapes where relatively few larval habitats (i.e. on the order of ten habitats per km^2^) are dispersed among people’s homes [16, 17]. If larval *Anopheles* habitats are considered foci of vector production [1], this relationship is intuitive. In this context, the gradient of adult *Anopheles* is at least partially determined by their dispersal distance, which then defines the focal extent. However, in regions where larval habitats are more abundant and distributed across the landscape, there may be multiple overlapping foci of vector production. Here, the relationship between larval habitat locations and the spatial distribution of adult *Anopheles* is less obvious because, for example, the density of habitats near a house may be as important, or more important, than the distance to the nearest habitat [18].

Public health interventions aimed at reducing malaria transmission also influence the spatial distribution of malaria. Insecticide-treated bed nets (ITNs), one of the primary vector control measures used since the 1990s, have a significant impact on malaria vectors, reducing their population sizes on a broad scale [19, 20]. At the household scale, variation in ITN ownership and use may lead to variation in the number of adult *Anopheles* found indoors. The presence of ITNs in houses may reduce the rate of entry [21], or increase the rate of exiting [21, 22], by adult *Anopheles*. Despite the substantial increase in the number of households owning ITNs in many malaria endemic countries over the last decade [23], variation in ITN use by individuals within households leaves some people unprotected [24–26]. Quantifying the contribution of these interventions to the heterogeneity of malaria, relative to that of landscape factors such as the distribution of larval *Anopheles* habitats, is critical for the effective implementation and evaluation of interventions.

The primary objective of this study was to quantify the contribution of the proximity of larval habitats to variation in the abundance of malaria vector species resting in houses, within a landscape where larval habitats are numerous yet heterogeneously distributed. Because of the relatively high proportion of households owning long-lasting insecticidal nets (LLIN) in the study site and the importance of LLINs as a malaria intervention, the effect of house-level LLIN use on the abundance of malaria vector species resting in houses was also quantified while accounting for variation in the proximity of the houses to larval habitats.

## Methods

### Study site

**Fig. 1:**
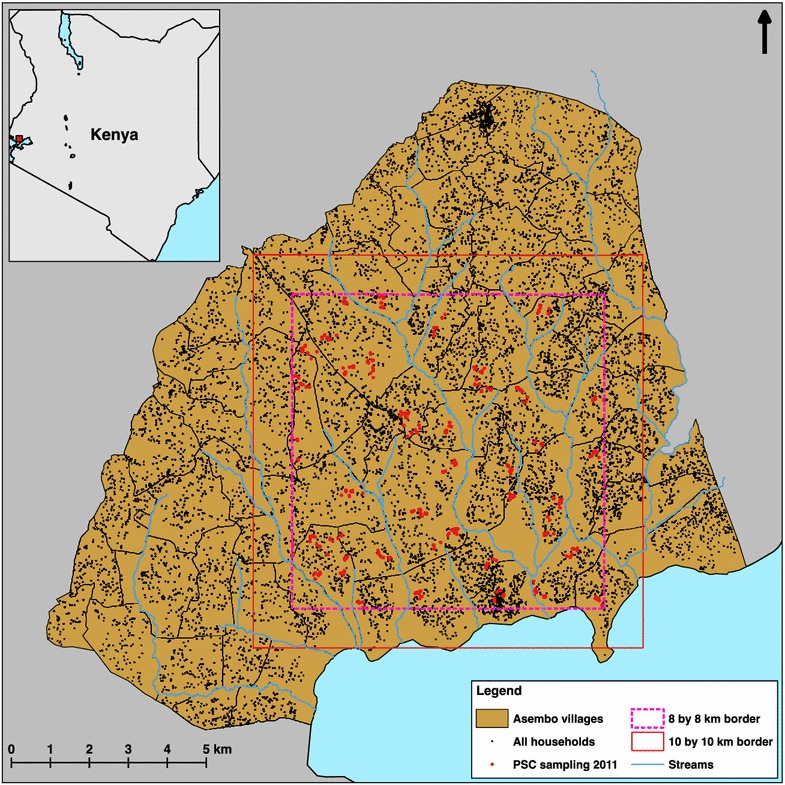
Study site in western Kenya. *Inset* shows Kenya with *small red square* indicating location of the study region in western Kenya. The *larger map* shows the boundaries of 76 villages in Asembo and the seasonal streams within the community, with *black dots* showing the geolocations of all households in the community. The 8 by 8 km border shows the extent of pyrethrum spray catch sampling in this study, with *red dots* showing the 525 houses sampled for adult *Anopheles.* The 10 by 10 km border shows the extent of the larval habitat model described in “Methods”

The Asembo region of Rarieda District (part of Siaya County) in western Kenya (Fig. 1) is a rural community of about 60,000 people covering an area of about 200 km^2^. Most of the residents are subsistence farmers, with the landscape largely dominated by small-scale agriculture. Small plots of land generally surround family-based groups of houses, which are further arranged into villages. While the houses are highly dispersed within villages, the boundaries between the 79 villages in Asembo are often discernable only by residents [27]. This is apparent in Fig. 1, which shows the roughly 10,500 households georeferenced within Asembo as of 2009 [28, 29]. Asembo sits in the lowlands along the shores of Lake Victoria, with elevations ranging from 1100 to 1400 m above sea level and low topographic relief. Rainfall is seasonally bimodal but local convective events may occur year round.

Malaria transmission occurs throughout the year, but seasonal peaks of transmission occur in May–July and October–November, tending to follow rainfall patterns with a short time lag. The predominant species of malaria is *Plasmodium falciparum*. Parasitaemia rates in children under 5 were around 40% in 2009 [30], although entomological inoculation rates have been estimated at less than 15 infectious bites per person per year since 2003 (MN Bayoh, unpublished data). Three primary malaria vector species are found in the region: *Anopheles funestus*, *Anopheles gambiae* s.s. and *Anopheles arabiensis* [31–34]. Larval *Anopheles* habitats are numerous and widespread in Asembo, but they are heterogeneously distributed as patches of varying size and may surround a house from multiple directions [35, 36]. Household-level ownership of ITNs and LLINs has been relatively high in Asembo (i.e. over 80%) since it was the site of a randomized, controlled trial of ITNs from 1997 to 1999, followed by periodic distribution of nets through national programmes [27, 33].

### Adult mosquito sampling

Houses were sampled for indoor-resting adult *Anopheles* between 16 May and 24 June 2011 using the pyrethrum spray catch method (PSC) [19, 37]. This period was chosen to coincide with seasonal peaks of *Anopheles* populations, which occur after the March–May rainy season [31, 32, 38]. Weather data for the period of this study were downloaded from the National Climatic Data Center’s Global Summary of Day (GSoD) database, using records of temperature and precipitation from a weather station at the Kisumu Airport (about 40 km east of Asembo). For missing data (10 out of 211 days for precipitation, 2 out of 211 days for temperature), the inverse distance weighted mean of surrounding GSoD weather stations (within 250 km) was used.

All *Anopheles* collected during PSC sampling were morphologically identified according to Gillies and Coetzee [39]. *Anopheles gambiae* s.s. and *An. arabiensis* were identified by PCR [40]. The heads and thoraces of all female *Anopheles* were tested for *P. falciparum* circumsporozoite proteins by ELISA [41] using the *P. falciparum* sporozoite ELISA reagent kit (MRA-890, MR4, ATCC, Manassas, VA, USA). The blood meal hosts of all fed and half-gravid female *Anopheles* were identified by direct sequencing of the vertebrate mitochondrial cytochrome B gene [42].

Houses for PSC mosquito sampling were selected from within an 8 by 8 km study area (Fig. 1) to avoid edge effects when assessing the effect of larval habitat proximity (described below) on the number of adult *Anopheles* collected. Houses were selected for sampling using two-stage cluster sampling. First, the 8 by 8 km area was divided into 1 km^2^ quadrats. Forty of these quadrats were randomly selected, and one house in each quadrat was randomly selected as the starting point for cluster sampling in that quadrat (i.e. 40 clusters of houses were sampled). Cluster sampling order was also randomly determined. On a given day of mosquito sampling, two teams of three field assistants each started at 0700 h by locating the selected house for their respective quadrat that day. After sampling at the selected house, the field team proceeded to sample at successive nearest-neighbour houses until 1000 h. In practice, the teams were able to sample from ten to twenty houses per cluster.

House-level variables that potentially influenced the number of *Anopheles* in the sampled houses were assessed at the time of mosquito sampling through a visual inspection of the house and via a standardized survey. The first of these was LLIN use. Ownership of LLINs was assessed through visual inspection, while use was self-reported during the survey. Specific numbers of LLIN users were counted in the surveys, and houses were later categorized into three groups of LLIN use: (a) houses where everyone who slept in the house the previous night used an LLIN; (b) houses where some residents had slept under an LLIN the previous night while other residents had not; (c) houses where no one had slept under an LLIN the previous night. Five additional house-level variables that potentially influenced the number of *Anopheles* in houses were included in the analyses based on findings from previous studies: presence of cattle near the house the previous night [16]; whether the inhabitants had cooked in the house the previous night [43, 44]; different wall types [15, 45, 46]; different roof types [15, 46]; and the number of people sleeping in the house the previous night [17, 45]. The status of the eaves (the gap between the top of the wall and the over-hanging roof) was also recorded as either open or closed [47, 48], but this variable was not included in any analyses, because fewer than 5% of sampled houses had closed eaves.

### Larval habitats

**Fig. 2:**
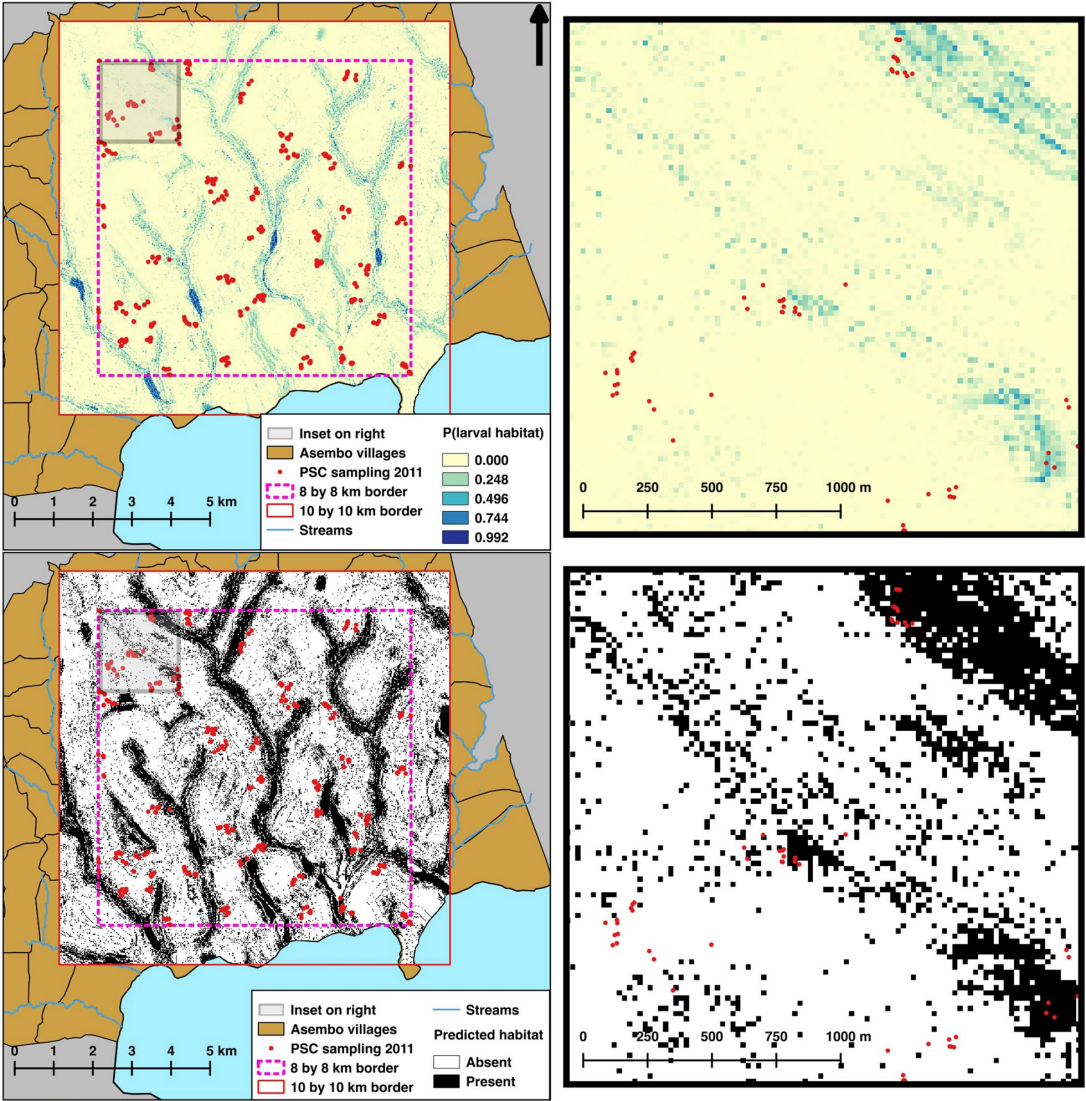
Predictive model of *Anopheles* larval habitat presence. The *top two panels* show the output of the predictive model as the probability (P) of a larval habitat being present in each 20 by 20 m pixel. The *bottom two panels* show the probabilities converted to either “present” (i.e. at least one larval habitat is expected to be present in the 20 by 20 m pixel) or “absent”, using a threshold of P = 0.020. Maps on the *right* show close-up views of the maps on the* left*

To characterize the relative juxtaposition of larval habitats to the houses sampled for adult mosquitoes, a predictive model of larval habitat locations was built as described in detail elsewhere [36], and model outputs (Fig. 2) were used to calculate several habitat proximity indices. First, the locations of larval *Anopheles* habitats were recorded in exhaustive ground surveys of thirty-one 500 by 500 m quadrats from 17 May to 4 July 2011. The surveyed quadrats were randomly selected, after spatial stratification, from a 10 by 10 km area within Asembo. Larval *Anopheles* habitats were defined as any standing body of water falling under the following habitat types: drainage channel, burrow pit, rain pool, runoff, cluster of hoof prints, stream bed pool, pond/reservoir, wet meadow, well and tire track [49]. The presence or absence of any *Anopheles* larvae was noted for each habitat, but all habitats were included in the final model regardless of whether *Anopheles* were present on the day of the ground survey. For a subset of habitats (up to 25 habitats per quadrat), *Anopheles* larvae and pupae were collected to confirm the presence of malaria vector species. All visible *Anopheles* larvae, up to a maximum of 20 per habitat, were collected using a 300 ml dipper or plastic pipette as appropriate according to the size of the habitat. The specimens were transported to the laboratory for species identification. Larvae were raised to fourth-stage instars for identification, while pupae were allowed to eclose as adults before identification. All identifications were done according to Gillies and Coetzee [39].

Using a topographic wetness index (TWI), land use/land cover (LULC), soil type, and distance to stream, the random forest statistical method [50] was applied to produce a landscape model of the probability of larval habitat presence at a 20 m resolution across the 10 by 10 km area [36]. A digital elevation model (DEM) of the study site was used to derive the TWI data. The 20 m resolution DEM was created using local universal kriging to interpolate 11,130 GPS elevation records previously taken within Asembo [28, 29]. The TWI data were then calculated using the ArcGIS extension TauDEM 5.0 (Tarboton, Utah State University). A satellite image from the IKONOS-2 sensor taken on 19 June 2007 was used to create the LULC classification at a 4 m resolution. Unsupervised classification was done using the K-means method [51] in ENVI 4.8 (Exelis Visual Information Solutions, Boulder, CO, USA). Classes were combined into a binary data layer of agricultural or non-agricultural land use. Soil data were taken from the 1:1,000,000 exploratory soil map of Kenya, compiled by the Kenya Soil Survey in 1980 [52]. All streams in Asembo were mapped using GPS units, and the Euclidean distance in metres to the nearest stream was calculated for each 20 by 20 m pixel of the 10 by 10 km area. The performance of the final model using these four variables was assessed by comparing the model output to holdout ground survey data, and calculating the area under the curve (AUC), sensitivity, specificity, percent correctly classified (PCC), and kappa.

This landscape model was used as a proxy for actual larval habitats locations in two ways (Fig. 2). First, the value at each 20 by 20 m pixel of the 10 by 10 km area was set equal to the probability of habitat presence as predicted by the model, and could take the value of any real number between 0 and 1. Second, the probabilities predicted by the model were converted to a binary value of either present (when P ≥ 0.020) or absent (when P < 0.020). Standard methods for converting modelled probabilities to a binary variable include using a threshold probability value (i.e. the value at which a probability equals presence rather than absence) that maximizes the agreement between observed locations (i.e. data collected for testing the model predictions) and locations predicted by the model, rather than simply using a fixed value (e.g. P = 0.5) [53]. In a previous study, a threshold value of P = 0.020 for the probability surface described above maximized this agreement [36].

Using these two outputs from the predictive model (probability of a habitat and presence/absence of a habitat) and the recorded geolocations of houses sampled for adult mosquitoes, the proximity of the houses to larval *Anopheles* habitats was quantified by calculating several proximity indices (Table 1). The first of these was the distance from the house to the nearest 20 by 20 m pixel predicted to have a habitat present in the binary output (i.e. distance to nearest habitat). Next, the number of pixels predicted to have a habitat (according to the binary output) within 50, 100, 300, 500 and 1000 m of the house was counted (i.e. number of habitats within *n* metres). Using the probability output, the mean value of all 20 by 20 m pixels within 50, 100, 300, 500 and 1000 m of the house was calculated (i.e. mean probability of a habitat within *n* metres). Both the minimum and maximum values for any one pixel within 50, 100, 300, 500 and 1000 m of the house were also extracted. Due to the high number of pixels with P = 0.000 across this landscape, all houses had at least one pixel with P = 0.000 within 300 m. Therefore, the minimum probability of a habitat within 300, 500, and 1000 m of a house was not used in further analysis. The range of distances over which to calculate the proximity indices was determined by assuming the average dispersal distance of *Anopheles* mosquitoes to be a few hundred metres while recognizing that it likely varies according to landscape patterns and human population density [1].

**Table 1:** Larval habitat proximity indices calculated for houses where sampling occurred for *Anopheles* mosquitoes

Proximity index	Measured at
Distance to nearest habitat^a^	NA
Number of habitats^a^ within n metres	50, 100, 300, 500, 1000 m
Maximum probability^b^ of a habitat within n metres	50, 100, 300, 500, 1000 m
Mean probability^b^ of a habitat within n metres	50, 100, 300, 500, 1000 m
Minimum probability^b^ of a habitat within n metres	50, 100^c^

*NA* not applicable

^a^Habitat locations were predicted using a random forest model, converting probabilities to presence and absence based on an empirically derived threshold of 0.020

^b^Probability of larval habitat presence was predicted using a random forest model

^c^The minimum probability of a habitat within a distance of ≥300 m of a house was 0 for all houses, and therefore not used in the analysis

### Statistical analysis

Regression with generalized linear models was used to quantify the association of the explanatory variables with the number of adult mosquitoes in houses, with separate analyses for each sex of *An. arabiensis*, *An. gambiae* s.s. and *An. funestus*. The explanatory variables were LLIN use, presence of cattle near the house the previous night, whether the inhabitants had cooked in the house the previous night, wall type, roof type, the number of people sleeping in the house the previous night, and a larval habitat index. As it was not clear a priori which of the larval habitat proximity indices described above would be associated with variation in adult Anopheles abundances, the larval habitat proximity index to use in each of the multivariate analyses was first determined by comparing univariate models with each of the habitat proximity indices to each other using Akaike information criteria (AIC). In the multivariate analyses, the single habitat proximity index from the univariate model with the lowest AIC for each respective sex and species was used. The presence of residual spatial correlation in the data was examined using the empirical variogram [54], on the Pearson’s residuals from a standard multivariate Poisson regression. In the final multivariate analyses, geostatistical Poisson regression with log-link functions was used to model the mosquito counts [55]. More specifically, the mean number of mosquitoes, $\mu (x)$, at house location *x* was modelled as

$$\log \{ \mu (x)\} = d(x)^{\prime}\beta + S(x) + Z(x),$$

where: $d(x)$ is a vector of explanatory variables; $S(x)$ is a random effect that accounts for the spatial correlation between houses induced by unmeasured factors affecting mosquito abundance; and $Z(x)$ is an unstructured random effect that accounts for extra-Poisson variation within houses. By including $S(x)$ in the model, we explicitly do not assume independence among observations. In this case, $S(x)$ was modelled as a Gaussian process with an isotropic Matern covariance function with variance parameter, *σ* ^*2*^, scale parameter, $\varphi$ (the distance beyond which the spatial correlation is below 0.05), and shape parameter, $\kappa$. Parameter estimation was done using Monte Carlo maximum likelihood, implemented in the PrevMap package [56] in the R software environment [57]. All maps presented were created using QGIS 2.14 [58]. All other figures, and all analyses, were implemented in R 3.3.2 [57].

## Results

**Fig. 3:**
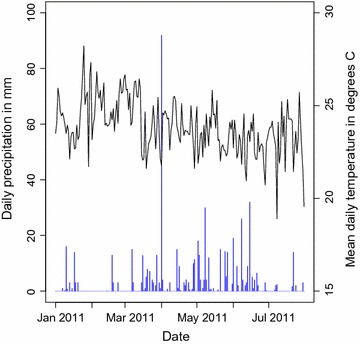
Weather data prior to and during study. The daily total precipitation (*blue bars*) and daily mean temperature (*black line*) from 1 January to 31 July 2011 at the Kisumu Airport weather station

**Fig. 4:**
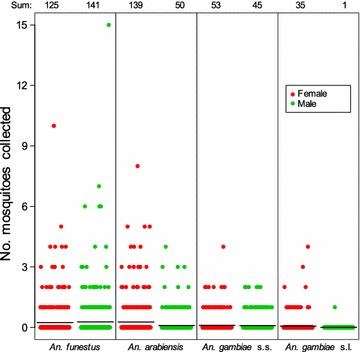
Number of mosquitoes collected per house by species and sex. *Each dot* within a species by sex represents a sample by PSC at one house. *Black horizontal lines* show the mean of each species by sex. Only those specimens identified morphologically as *An. gambiae* species complex and not identified further by PCR are counted for *An. gambiae* s.l. The sum total for each species by sex is shown at the *top*

Daily total precipitation and daily mean temperature were normal for the region and season during the study period (Fig. 3). Totals of 356 female *Anopheles* and 241 male *Anopheles* were collected in the 525 houses sampled. Means of 0.24 *An. funestus* females, 0.26 *An. arabiensis* females and 0.10 *An. gambiae* s.s. females were collected per house (Fig. 4). Totals of 4 female and 4 male *Anopheles rufipes* were also collected. Nearly 70% of the houses (n = 358) did not have any *Anopheles* females on the day of sampling. The *An. gambiae* s.l. specimens consisted of 59% *An. arabiensis* (139 females and 50 males), 30% *An. gambiae* s.s. (53 females and 45 males) and 11% which were not identified further (35 females and 1 male). The sporozoite rate for all species combined was 4% (Table 2). Most of the *Anopheles* females were either fed (58%), half-gravid (13%) or gravid (21%) (Table 2). Based on the 163 *Anopheles* females for which blood meal hosts were successfully identified, *An. funestus* and *An. gambiae* s.s. fed almost exclusively on humans, and *An. arabiensis* fed on human, cattle and goat (Table 2).

**Table 2:** Summary of female *Anopheles* mosquitoes collected during PSC sampling

	Number of females (%)					
	*An. funestus*	*An. arabiensis*	*An. gambiae* s.s.	*An. gambiae* s.l.^a^	*An. rufipes*	Total
Pf sporozoite ELISA						
Negative	116 (0.93)	138 (0.99)	49 (0.92)	34 (0.97)	4 (1.00)	341 (0.96)
Positive	9 (0.07)	1 (0.01)	4 (0.08)	1 (0.03)	0 (0.00)	15 (0.04)
Abdominal status						
Unfed	15 (0.12)	6 (0.04)	5 (0.09)	1 (0.03)	0 (0.00)	27 (0.08)
Gravid	31 (0.25)	35 (0.25)	9 (0.17)	1 (0.03)	0 (0.00)	76 (0.21)
Half-gravid	15 (0.12)	17 (0.12)	7 (0.13)	7 (0.20)	0 (0.00)	46 (0.13)
Fed	63 (0.50)	81 (0.58)	32 (0.60)	26 (0.74)	4 (1.00)	206 (0.58)
Not available	1 (0.01)	0 (0.00)	0 (0.00)	0 (0.00)	0 (0.00)	1 (0.00)
Blood meal host						
Human	63 (0.81)	11 (0.11)	30 (0.77)	5 (0.15)	2 (0.50)	111 (0.44)
Cattle	2 (0.03)	30 (0.31)	1 (0.03)	14 (0.42)	1 (0.25)	48 (0.19)
Goat	0 (0.00)	2 (0.02)	0 (0.00)	1 (0.03)	1 (0.25)	4 (0.02)
No amplicon	6 (0.08)	28 (0.29)	4 (0.10)	4 (0.12)	0 (0.00)	42 (0.17)
Not done	7 (0.09)	27 (0.28)	4 (0.10)	9 (0.27)	0 (0.00)	47 (0.19)

Pf, *Plasmodium falciparum*

^a^These specimens were identified morphologically as *An. gambiae* species complex but not identified further by PCR

In the 31 quadrats where ground surveys were conducted, 1673 larval *Anopheles* habitats were observed, with a mean of 54 habitats observed per 500 by 500 m quadrat (range 0–303). *Anopheles* larvae were present in 921 of the 1673 habitats on the day each habitat was recorded. *Anopheles* larvae and pupae were identified from 141 of the habitats, 77% of which were occupied by *An. gambiae* s.l. on the day of collection. Most of the larvae and pupae were identified as *An. gambiae* s.l. (79%). The other species collected were *An. funestus* (1.1%), *Anopheles coustani* (6.7%), *Anopheles rufipes* (5.3%), *Anopheles maculipalpis* (2.5%) and *Anopheles pharoensis/squamosus* (3.9%). The predictive landscape model for larval *Anopheles* habitats had an AUC of 0.808, sensitivity of 0.750, specificity of 0.725, PCC of 0.726 and kappa of 0.145.

**Fig. 5:**
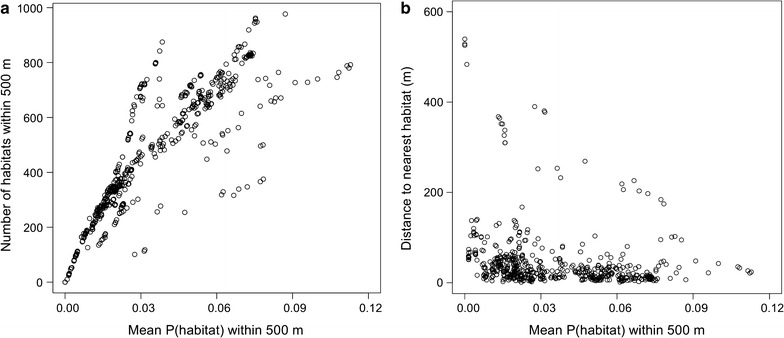
Scatterplots comparing two different larval habitat indices (**a** number of habitats within 500 m; **b** distance to nearest habitat in metres) with the mean probability of a habitat within 500 m. *Each dot* represents one of the 525 houses sampled in this study. P (habitat), probability of a larval habitat

Houses varied considerably in their proximity to larval *Anopheles* habitats. Distance from the sampled houses to the nearest predicted larval habitat ranged from 1 to 539 m (mean = 48 m). The number of predicted larval habitats within 500 m of the sampled houses ranged from 0 to 977 (mean = 462), and the mean probability of larval habitat presence within 500 m ranged from 0.0 to 11.3% (mean = 3.5%). Some of the larval habitat proximity indices were highly correlated with each other. For example, the mean probability of larval habitat presence within 500 m of a house was more strongly correlated with the number of habitats within 500 m of a house (r = 0.90; Fig. 5a) than the distance to the nearest larval habitat (r = −0.37; Fig. 5b).

The larval habitat proximity index that best fit the observed variation in female *An. arabiensis* collected per house was the mean probability of larval habitat presence within 500 m of a house (Table 3). Based on AIC weight [59], there was also support for considering the number of habitats within 500 m of a house as an explanatory variable for variation in *An. arabiensis* females (Additional file 1), but only the mean probability of larval habitat presence was used in the full geostatistical regression model because of the high correlation between the two variables. For male *An. arabiensis*, the larval habitat proximity index that best fit the observed data was the maximum probability of larval habitat presence within 50 m of a house (Table 3). However, there was also some support for at least 5 of the other habitat indices according to AIC, including the number of habitats within 50 m, the number of habitats within 300 m and the mean probability of larval habitat presence within 300 m (Additional file 1). For *An. funestus* females, the mean probability of larval habitat presence within 1000 m of a house best fit the observed variation (Table 3), though there was also support for considering the maximum probability of larval habitat presence within 1000 m based on AIC weight (Additional file 1). *Anopheles funestus* males responded similarly to *An. arabiensis* females on this landscape (Table 3), except the number of habitats within 500 m had a lower AIC than the mean probability of larval habitat presence within 500 m (Additional file 1). The selection of a habitat index for both female and male *An. gambiae* s.s. was less clear, as none of the model weights [59] was higher than 0.154 (Additional file 1). In the full geostatistical regression models for female and male *An. gambiae* s.s., the mean probability of larval habitat presence within 300 m and the maximum probability of larval habitat presence within 100 m were used, respectively (Table 3).

**Table 3:** Larval habitat proximity index (LHPI) used in each full geostatistical model

Species	Sex	LHPI used in full geostatistical model
*An. arabiensis*	Female	Mean P (habitat) within 500 m
*An. arabiensis*	Male	Maximum P (habitat) within 50 m
*An. gambiae* s.s.	Female	Mean P (habitat) within 300 m
*An. gambiae* s.s.	Male	Maximum P (habitat) within 100 m
*An. funestus*	Female	Mean P (habitat) within 1000 m
*An. funestus*	Male	Number of habitats within 500 m

The larval habitat proximity index used in the full geostatistical Poisson regression model for each species by sex, as determined by comparing single-covariate models using each of the calculated larval habitat proximity indices

The number of adult female *An. arabiensis* per house increased considerably with increasing mean probability of larval habitat presence within 500 m (Table 4). A similar effect was observed for both sexes of all three species, in that higher numbers of adult *Anopheles* were associated with higher values of each respective larval habitat proximity index.

**Table 4:** Relative rate ratios of *Anopheles* per house, according to multi-covariate geostatistical Poisson regression models

Variable (n)	Relative rate ratio (95% CI)					
	*An. arabiensis*		*An. gambiae* s.s.		*An. funestus*	
	Females	Males	Females	Males	Females	Males
*Habitat index* ^a^						
1	1.00	1.00	1.00	1.00	1.00	1.00
2	0.85 (0.79, 0.93)	0.98 (0.92, 1.04)	0.69 (0.65, 0.72)	1.11 (1.06, 1.15)	2.01 (1.49, 2.72)	1.40 (1.27, 1.53)
3	1.79 (1.54, 2.08)	0.96 (0.90, 1.04)	0.53 (0.50, 0.57)	1.13 (1.08, 1.18)	1.23 (0.85, 1.76)	4.25 (3.50, 5.16)
4	2.24 (1.89, 2.65)	1.75 (1.62, 1.90)	1.38 (1.26, 1.51)	1.35 (1.27, 1.42)	2.30 (1.57, 3.37)	4.66 (3.78, 5.74)
*LLIN use*						
None (130)	1.00	1.00	1.00	1.00	1.00	1.00
Some (81)	0.84 (0.82, 0.86)	1.97 (1.91, 2.04)	0.87 (0.85, 0.89)	0.88 (0.87, 0.90)	0.38 (0.37, 0.40)	0.91 (0.88, 0.94)
All (314)	0.49 (0.48, 0.50)	0.50 (0.48, 0.51)	0.39 (0.39, 0.40)	0.82 (0.81, 0.83)	0.60 (0.58, 0.61)	1.01 (0.99, 1.03)
*Cooked*						
No (342)	1.00	1.00	1.00	1.00	1.00	1.00
Yes (183)	0.26 (0.26, 0.27)	0.32 (0.31, 0.33)	0.47 (0.47, 0.48)	0.37 (0.36, 0.37)	0.57 (0.56, 0.59)	0.42 (0.42, 0.43)
*Cattle*						
No (166)	1.00	1.00	1.00	1.00	1.00	1.00
Yes (359)	2.39 (2.29, 2.50)	2.45 (2.34, 2.58)	2.76 (2.66, 2.85)	1.39 (1.34, 1.43)	0.88 (0.83, 0.94)	0.69 (0.66, 0.73)
*Roof*						
Thatch (161)	1.00	1.00	1.00	1.00	1.00	1.00
Iron (364)	1.25 (1.23, 1.28)	0.77 (0.75, 0.78)	1.21 (1.19, 1.23)	0.77 (0.76, 0.78)	0.63 (0.62, 0.65)	0.46 (0.45, 0.47)
*Wall*						
Mud (280)	1.00	1.00	1.00	1.00	1.00	1.00
Plastered (109)	0.80 (0.78, 0.82)	0.52 (0.51, 0.53)	0.80 (0.79, 0.82)	1.26 (1.24, 1.27)	1.67 (1.62, 1.73)	1.36 (1.33, 1.40)
Other (136)	0.72 (0.70, 0.74)	0.38 (0.37, 0.39)	1.15 (1.13, 1.17)	0.82 (0.81, 0.83)	0.66 (0.63, 0.68)	1.04 (1.01, 1.07)
*No. people*						
(Mean = 3)	0.87 (0.86, 0.87)	0.72 (0.71, 0.72)	1.10 (1.10, 1.11)	1.06 (1.06, 1.06)	1.15 (1.14, 1.16)	0.90 (0.89, 0.90)
*Geostatistical parameters*						
*σ* ^*2*^	0.27 (0.19, 0.38)	0.49 (0.32, 0.76)	0.14 (0.10, 0.19)	0.15 (0.10, 0.22)	1.27 (0.74, 2.20)	0.75 (0.45, 1.25)
$\varphi$	0.56 (0.38, 0.83)	0.84 (0.54, 1.31)	0.47 (0.33, 0.67)	0.83 (0.54, 1.26)	1.35 (0.77, 2.37)	1.21 (0.72, 2.05)

*LLIN* long-lasting impregnated nets. LLIN use, whether none, some or all of the residents used a LLIN the previous night; Cooked, whether residents cooked in the house the previous night; Cattle, whether cattle were present near the house the previous night; Roof, the main material of the roof; Wall, the main material of the walls; No. people, the number of people sleeping in the house the previous night; *σ* ^*2*^, variance parameter of the estimated spatial correlation; $\varphi$, scale parameter of the estimated spatial correlation

^a^All analyses were done with larval habitat indices as continuous variables, but they are presented here as factors (grouped by quartiles within each habitat index) for a more intuitive interpretation of the effect on the relative rate ratio. The habitat index used for each species and sex is listed in Table 3. A higher value for habitat index indicates a house is more likely to be closer to more larval habitats

House-level variables (i.e. LLIN use, the presence of cattle, whether the inhabitants had cooked in the house, different wall types, different roof types, and the number of people sleeping in the house the previous night) also contributed to variation in the number of *Anopheles* collected. House-level ownership of at least one LLIN was 75%. Still, only 60% of respondents reported that all residents in their house used an LLIN the previous night. Of the 25% of houses in which none of the residents slept under an LLIN the previous night, none had an LLIN present. These houses without an LLIN had higher abundances of all three species than houses in the other two LLIN categories, in which some or all of the residents used an LLIN the previous night; furthermore, there were lower abundances of *An. arabiensis* and *An. gambiae* s.s. inside houses where all of the residents used an LLIN the previous night compared to houses where only some of the residents used an LLIN (Table 4).

Houses with cattle nearby had more *An. arabiensis* and *An. gambiae* s.s., but fewer *An. funestus* (Table 4). The number of *An. arabiensis* decreased with increasing number of people sleeping in the house, but the number of *An. gambiae* s.s. and *An. funestus* was positively associated with the number of people (Table 4). A lower abundance of all three species was associated with cooking in the house the previous night (Table 4).

The empirical variograms from the standard multivariate Poisson regression models indicated residual spatial correlation in the data. The scale of spatial autocorrelation ($\varphi$) estimated in the geostatistical regression models varied among the six species-sex combinations. Models for *An. arabiensis* females and *An. gambiae* s.s. females had the lowest scale of spatial autocorrelation (about 500 m), while males of those same two species had an estimated scale of spatial autocorrelation around 800 m (Table 4). For both sexes of *An. funestus* the scale of spatial autocorrelation was about 1200 m.

## Discussion

The proximity of houses to larval *Anopheles* habitats contributes significantly to the indoor resting abundance of adult malaria vector species in this region where larval habitats are numerous and heterogeneously distributed. This agrees broadly with findings in other landscapes [6, 15, 18], but there was also variation in the statistical fit of different larval habitat proximity indices (e.g. distance to nearest habitat compared to mean probability of a habitat within 500 m), suggesting that the relationship between larval habitats and the distribution of adult *Anopheles* may not depend on simply the distance to the nearest habitat in all landscapes. Previous studies have largely been conducted in landscapes where there is a close association between the distance to the nearest larval habitat and the number of larval habitats near a house (e.g. all houses with shorter distances to the nearest larval habitat have a similar number of larval habitats nearby when most larval habitats are found along a linear feature such as a stream bed) [6]. In those landscapes, using distance to the nearest habitat and using the number of nearby habitats to explain the number of adult *Anopheles* in houses would give similar results. In contrast, most houses in this study were less than 200 m from the nearest predicted larval habitat, yet indices related to the density of larval habitats near those houses varied more than, and was mostly unrelated to, the distance to the nearest habitat (Fig. 5b). Similar to a study in The Gambia [18], the statistical models in this study using indices related to the density of larval habitats generally fit the observed data better than simply measuring the distance to the nearest single larval habitat. Still, the distribution of adult *Anopheles* is clearly associated with the locations of larval habitats in a broad sense, suggesting that *An. arabiensis*, *An. gambiae* s.s. and *An. funestus* preferentially disperse relatively short distances when their required resources are readily available. Presumably, this relates to the energetic costs of flight [60, 61].

Variation in LLIN use among houses also contributed to variation in the number of adult *Anopheles* found in this study. In the sampled houses, all survey respondents owning at least one LLIN within the house reported at least one person sleeping under an LLIN the previous night. Still, 25% of houses did not own any LLIN, and at least one person did not sleep under a LLIN in a further 15% of the sampled houses. High community-level coverage of ITNs reduces the abundance of malaria vector populations and the risk of malaria morbidity and mortality, even in houses not owning any ITN [19, 62]. Despite this community-wide benefit, differences remain in the abundance of all malaria vector species between houses with and without ownership of at least one LLIN. Furthermore, houses in which only some of the residents used LLINs the previous night also had more *An. arabiensis* and *An. gambiae* s.s. than houses in which all of the residents used LLINs the previous night, an effect which was also seen during the ITN trial in this area in 1997–1999 [19]. A likely explanation is that people not sleeping under LLINs, even in houses with LLINs, represent potential hosts for *Anopheles* females. These differences among houses in *Anopheles* abundance probably explain the observed difference in malaria risk attributed to LLIN use in other areas of high LLIN ownership [63, 64].

In addition to larval habitat proximity and LLIN use, the other house-level variables measured here accounted for variation among sampled houses in the number of *Anopheles* collected indoors. For example, cooking in the house the previous night was associated with collecting fewer mosquitoes of all three species and both sexes, which may be attributed to the smoke produced while cooking. Wood and charcoal are the predominant fuels for cooking in Asembo, and the smoke from firewood may reduce the number of *Anopheles* found indoors [44]. However, this may be due to increased house-exiting behaviour as opposed to a true repellency effect. Biran and colleagues [65] found little evidence for a protective effect of smoke from domestic fires against mosquitoes in their systematic review, noting that three observational studies from the early twentieth century in South and East Africa [66–68] found no difference in the numbers of *Anopheles* between homes with and without smoke from domestic fires. Furthermore, the increased risk of respiratory diseases linked to smoke from biofuel sources [69] may outweigh any potential decrease in malaria risk due to a reduced number of *Anopheles* indoors.

This study examined the determinants of variation in *Anopheles* adults in houses during the yearly peak in population size. Clearly, *Anopheles* populations in the region vary seasonally [19, 31, 32, 38], and the relationships found here may change in magnitude or even direction with the seasons. Additionally, variation among years in precipitation patterns could influence the relationships found here. The advantage to this cross-sectional approach was our ability to cover a relatively large area, capturing greater variation in the landscape and potentially making the results more generalizable.

**Fig. 6:**
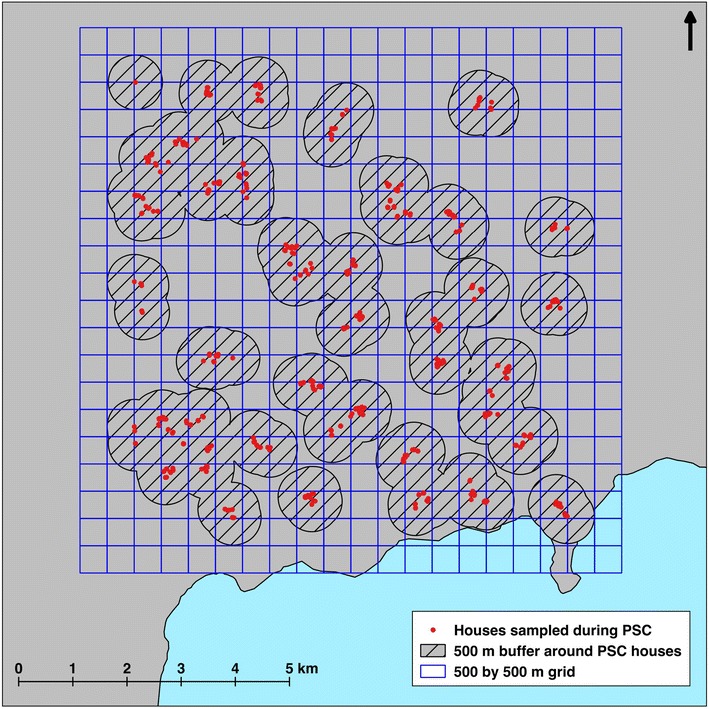
Area required for full larval habitat surveys compared to modelling. A *circle* (or buffer) with 500 m radius is drawn around the geolocation of each house sampled during pyrethrum spray catch sampling. Overlapping buffers are combined (or dissolved) with one another before calculating the total area required for ground surveys of larval *Anopheles* habitats. The 10 by 10 km study site is shown as a 500 by 500 m grid, from which 31 quadrats were actually surveyed to build the model used in this study

Using ground surveys to identify all larval *Anopheles* habitats potentially contributing to adult mosquito abundance in the sampled houses for this study was considered impractical. The ground surveys conducted to produce the predictive landscape model of potential habitats covered a total of 775 hectares. Ground surveys to identify larval *Anopheles* habitats within 500 m of all 525 houses sampled in this study would have required covering 4318 ha, or more than five times the area actually covered (Fig. 6). The results presented here demonstrate the utility of linking ground surveys of randomly selected habitats and households with remotely sensed data to develop models for predicting areas of high malaria transmission and for potentially identifying areas to target with larval source management.

## Conclusions

Understanding the ecological drivers of fine-scale spatial heterogeneity in malaria vector abundance is essential for the design, implementation and continued evaluation of appropriate malaria interventions, especially as control programmes work toward transmission reduction and elimination [70, 71]. Despite sustained, high coverage of ITNs and relatively high rates of ITN use, malaria persists in this region of western Kenya. A feature of this phenomenon, characterized as persistent residual transmission [72], is low vector density and low transmission intensity. In this study, household vector abundance was low but malaria transmission continues [30], a condition that has been observed for over a decade in this region [73]. Still, variation in house-level malaria vector abundance was attributed to both LLIN use and proximity to larval *Anopheles* habitats. This suggests that further increases in LLIN use, if possible to achieve, would lead to further individual-level protection for those new LLIN users. It also suggests that integrating effective larval source management as an additional component of the malaria control programme in the region would lead to further reductions in malaria risk by reducing the amount of an obligatory resource for mosquitoes (i.e. suitable aquatic habitat for immature stages) near people’s homes.

## Additional file

**Additional file 1.** Comparison of model fits for univariate models with each of the larval habitat proximity indices.

## Abbreviations

AIC: akaike information criteria
AUC: area under the curve
DEM: digital elevation model
ITN: insecticide-treated net
LLIN: long-lasting insecticidal net
LULC: land use/land cover
PCC: percent correctly classified
TWI: topographic wetness index
PSC: pyrethrum spray catch

## References

[1] Carter R, Mendis KN, Roberts D (2000). Spatial targeting of interventions against malaria. Bull World Health Organ.

[2] Boyd MF, Boyd MF (1949). Historical overview. Malariology: a comprehensive survey of all aspects of this group of diseases from a global standpoint.

[3] Greenwood BM (1989). The microepidemiology of malaria and its importance to malaria control. Trans R Soc Trop Med Hyg.

[4] Gamage-Mendis AC, Carter R, Mendis C, De Zoysa AP, Herath PR, Mendis KN (1991). Clustering of malaria infections within an endemic population: risk of malaria associated with the type of housing construction. Am J Trop Med Hyg.

[5] Mackinnon MJ, Gunawardena DM, Rajakaruna J, Weerasingha S, Mendis KN, Carter R (2000). Quantifying genetic and nongenetic contributions to malarial infection in a Sri Lankan population. Proc Natl Acad Sci USA.

[6] Trape J-F, Lefebvre-Zante E, Legros F, Ndiaye G, Bouganali H, Druilhe P (1992). Vector density gradients and the epidemiology of urban malaria in Dakar, Senegal. Am J Trop Med Hyg..

[7] Thompson RR, Begtrup KK, Cuamba NN, Dgedge MM, Mendis CC, Gamage-Mendis AA (1997). The Matola malaria project: a temporal and spatial study of malaria transmission and disease in a suburban area of Maputo, Mozambique. Am J Trop Med Hyg..

[8] Ribeiro JMC, Seulu F, Abose T, Kidane G, Teklehaimanot A (1996). Temporal and spatial distribution of anopheline mosquitos in an Ethiopian village: implications for malaria control strategies. Bull World Health Organ.

[9] Ghebreyesus TA, Haile M, Witten KH, Getachew A, Yohannes AM, Yohannes M (1999). Incidence of malaria among children living near dams in northern Ethiopia: community based incidence survey. BMJ.

[10] Clark TD, Greenhouse B, Njama Meya D, Nzarubara B, Maiteki Sebuguzi C, Staedke SG (2008). Factors determining the heterogeneity of malaria incidence in children in Kampala, Uganda. J Infect Dis.

[11] Oesterholt MJAM, Bousema JT, Mwerinde OK, Harris C, Lushino P, Masokoto A (2006). Spatial and temporal variation in malaria transmission in a low endemicity area in northern Tanzania. Malar J.

[12] Clarke SE, Bogh C, Brown RC, Walraven GEL, Thomas CJ, Lindsay SW (2002). Risk of malaria attacks in Gambian children is greater away from malaria vector breeding sites. Trans R Soc Trop Med Hyg.

[13] Smith MW, Macklin MG, Thomas CJ (2013). Hydrological and geomorphological controls of malaria transmission. Earth Sci Rev.

[14] van der Hoek W, Konradsen F, Amerasinghe PH, Perera D, Piyaratne MK, Amerasinghe FP (2003). Towards a risk map of malaria for Sri Lanka: the importance of house location relative to vector breeding sites. Int J Epidemiol.

[15] Zhou G, Munga S, Minakawa N, Githeko AK, Yan G (2007). Spatial relationship between adult malaria vector abundance and environmental factors in western Kenya highlands. Am J Trop Med Hyg.

[16] Minakawa N, Seda P, Yan G (2002). Influence of host and larval habitat distribution on the abundance of African malaria vectors in western Kenya. Am J Trop Med Hyg.

[17] Walker M, Winskill P, Basáñez M-G, Mwangangi JM, Mbogo CM, Beier JC (2013). Temporal and micro-spatial heterogeneity in the distribution of *Anopheles* vectors of malaria along the Kenyan coast. Parasit Vectors.

[18] Bogh C, Lindsay SW, Clarke SE, Dean A, Jawara M, Pinder M (2007). High spatial resolution mapping of malaria transmission risk in the Gambia, west Africa, using LANDSAT TM satellite imagery. Am J Trop Med Hyg.

[19] Gimnig JE, Vulule JM, Lo TQ, Kamau L, Kolczak MS, Phillips-Howard PA (2003). Impact of permethrin-treated bed nets on entomologic indices in an area of intense year-round malaria transmission. Am J Trop Med Hyg.

[20] Gimnig JE, Kolczak MS, Hightower AW, Vulule JM, Schoute E, Kamau L (2003). Effect of permethrin-treated bed nets on the spatial distribution of malaria vectors in western Kenya. Am J Trop Med Hyg.

[21] Mathenge EM, Gimnig JE, Kolczak M, Ombok M, Irungu LW, Hawley WA (2001). Effect of permethrin-impregnated nets on exiting behavior, blood feeding success, and time of feeding of malaria mosquitoes (Diptera: Culicidae) in western Kenya. J Med Entomol.

[22] Malima RC, Magesa SM, Tungu PK, Mwingira V, Magogo FS, Sudi W (2008). An experimental hut evaluation of Olyset^®^ nets against anopheline mosquitoes after seven years use in Tanzanian villages. Malar J.

[23] WHO (2012). World malaria report 2012.

[24] Korenromp EL, Miller J, Cibulskis RE, Cham MK, Alnwick D, Dye C (2003). Monitoring mosquito net coverage for malaria control in Africa: possession vs. use by children under 5 years. Trop Med Int Health.

[25] Eisele TP, Keating J, Littrell M, Larsen D, Macintyre K (2009). Assessment of insecticide-treated bednet use among children and pregnant women across 15 countries using standardized national surveys. Am J Trop Med Hyg.

[26] Macintyre K, Littrell M, Hamainza B, Miller J, Eisele TP, Keating J (2012). Determinants of hanging and use of ITNs in the context of near universal coverage in Zambia. Health Policy Plan.

[27] Phillips-Howard PA, Nahlen BL, Alaii JA, ter Kuile FO, Gimnig JE, Terlouw DJ (2003). The efficacy of permethrin-treated bed nets on child mortality and morbidity in western Kenya I: development of infrastructure and description of study site. Am J Trop Med Hyg.

[28] Hightower AW, Ombok M, Otieno R, Odhiambo R, Oloo AJ, Lal AA (1998). A geographic information system applied to a malaria field study in western Kenya. Am J Trop Med Hyg.

[29] Ombok M, Adazu K, Odhiambo F, Bayoh MN, Kiriinya R, Slutsker L (2010). Geospatial distribution and determinants of child mortality in rural western Kenya 2002–2005. Trop Med Int Health.

[30] Hamel MJ, Adazu K, Obor D, Sewe M, Vulule JM, Williamson JM (2011). A reversal in reductions of child mortality in western Kenya, 2003–2009. Am J Trop Med Hyg.

[31] Beier JC, Perkins PV, Onyango FK, Gargan TP, Oster CN, Whitmire RE (1990). Characterization of malaria transmission by *Anopheles* (Diptera: Culicidae) in western Kenya in preparation for malaria vaccine trials. J Med Entomol.

[32] Taylor KA, Koros JK, Nduati J, Copeland RS, Collins FH, Brandling-Bennett AD (1990). *Plasmodium falciparum* infection rates in *Anopheles gambiae*, *An. arabiensis*, and *An. funestus* in western Kenya. Am J Trop Med Hyg.

[33] Bayoh MN, Mathias DK, Odiere MR, Mutuku FM, Kamau L, Gimnig JE (2010). *Anopheles gambiae*: historical population decline associated with regional distribution of insecticide-treated bed nets in western Nyanza Province, Kenya. Malar J.

[34] McCann RS, Ochomo E, Bayoh MN, Vulule JM, Hamel MJ, Gimnig JE (2014). Reemergence of *Anopheles funestus* as a vector of *Plasmodium falciparum* in western Kenya after long-term implementation of insecticide-treated bed nets. Am J Trop Med Hyg.

[35] Mutuku F, Bayoh MN, Hightower A, Vulule JM, Gimnig JE, Mueke J (2009). A supervised land cover classification of a western Kenya lowland endemic for human malaria: associations of land cover with larval *Anopheles* habitats. Int J Health Geogr.

[36] McCann RS, Messina JP, MacFarlane DW, Bayoh MN, Vulule JM, Gimnig JE (2014). Modeling larval malaria vector habitat locations using landscape features and cumulative precipitation measures. Int J Health Geogr.

[37] Silver JB (2008). Mosquito ecology: field sampling methods. Third.

[38] Odiere M, Bayoh MN, Gimnig JE, Vulule JM, Irungu L, Walker ED (2007). Sampling outdoor, resting *Anopheles gambiae* and other mosquitoes (Diptera: Culicidae) in western Kenya with clay pots. J Med Entomol.

[39] Gillies MT, Coetzee M (1987). A supplement to the Anophelinae of Africa south of the Sahara (Afrotropical Region).

[40] Scott JA, Brogdon WG, Collins FH (1993). Identification of single specimens of the *Anopheles gambiae* complex by the polymerase chain reaction. Am J Trop Med Hyg.

[41] Wirtz RA, Zavala F, Charoenvit Y, Campbell GH, Burkot TR, Schneider I (1987). Comparative testing of monoclonal antibodies against *Plasmodium falciparum* sporozoites for ELISA development. Bull World Health Organ.

[42] Hamer GL, Kitron UD, Brawn JD, Loss SR, Ruiz MO, Goldberg TL (2008). *Culex pipiens* (Diptera: Culicidae): a bridge vector of West Nile virus to humans. J Med Entomol.

[43] Hiscox A, Khammanithong P, Kaul S, Sananikhom P, Luthi R, Hill N (2013). Risk factors for mosquito house entry in the Lao PDR. PLoS ONE.

[44] Bockarie MJM, Service MWM, Barnish GG, Momoh WW, Salia FF (1994). The effect of woodsmoke on the feeding and resting behaviour of *Anopheles gambiae* s.s. Acta Trop.

[45] Kirby MJ, Green C, Milligan PM, Sismanidis C, Jasseh M, Conway DJ (2008). Risk factors for house-entry by malaria vectors in a rural town and satellite villages in The Gambia. Malar J.

[46] Lwetoijera DW, Kiware SS, Mageni ZD, Dongus S, Harris C, Devine GJ (2013). A need for better housing to further reduce indoor malaria transmission in areas with high bed net coverage. Parasit Vectors.

[47] Tusting LS, Ippolito MM, Willey BA, Kleinschmidt I, Dorsey G, Gosling RD (2015). The evidence for improving housing to reduce malaria: a systematic review and meta-analysis. Malar J.

[48] Njie M, Dilger E, Lindsay SW, Kirby MJ (2009). Importance of eaves to house entry by anopheline, but not culicine, mosquitoes. J Med Entomol.

[49] Mutuku FM, Alaii JA, Bayoh MN, Gimnig JE, Vulule JM, Walker ED (2006). Distribution, description, and local knowledge of larval habitats of *Anopheles gambiae* s.l. in a village in western Kenya. Am J Trop Med Hyg.

[50] Breiman L (2001). Random forests. Mach Learn.

[51] MacQueen J. Some methods for classification and analysis of multivariate observations. Proceedings of the fifth Berkeley symposium on mathematical statistics and probability; 1967. p. 281–97.

[52] Sombroek WG, Braun HMH, van der Pouw BJA. Exploratory soil map and agro-climatic zone map of Kenya, 1980. Scale 1:1,000,000. Nairobi: Kenya Soil Survey; 1982. Report No.: E1.

[53] Liu C, Berry PM, Dawson TP, Pearson RG (2005). Selecting thresholds of occurrence in the prediction of species distributions. Ecography.

[54] Bivand RS, Pebesma E, Gómez-Rubio V (2013). Interpolation and geostatistics. Applied Spatial Data Analysis with R.

[55] Bolker BM, Brooks ME, Clark CJ, Geange SW, Poulsen JR, Stevens MHH (2009). Generalized linear mixed models: a practical guide for ecology and evolution. Trends Ecol Evol.

[56] Giorgi E, Diggle PJ (2017). PrevMap: an R Package for Prevalence Mapping. J Stat Soft.

[57] R Core Team. R: a language and environment for statistical computing. Vienna, Austria: R Foundation for Statistical Computing; 2016. https://www.R-project.org. Accessed 8 Feb 2017.

[58] QGIS Development Team. QGIS geographic information system. Open Source Geospatial Foundation Project. http://qgis.osgeo.org. Accessed 8 Feb 2017.

[59] Burnham K, Anderson D. Model selection and multimodel inference: a practical information-theoretical approach, 2nd ed. In: Burnham KP, Anderson DR, editors. New York: Springer; 2002.

[60] Nayar JK, Van Handel E (1971). The fuel for sustained mosquito flight. J Insect Physiol.

[61] Foster WA (1995). Mosquito sugar feeding and reproductive energetics. Annu Rev Entomol.

[62] Hawley WA, Phillips-Howard PA, ter Kuile FO, Terlouw DJ, Vulule JM, Ombok M (2003). Community-wide effects of permethrin-treated bed nets on child mortality and malaria morbidity in western Kenya. Am J Trop Med Hyg.

[63] Wotodjo AN, Diagne N, Gaudart J, Richard V, Raoult D, Sokhna C (2015). Malaria risk factors in Dielmo, a Senegalese malaria-endemic village, between October and November of 2013: a case-control study. Am J Trop Med Hyg.

[64] Okoyo C, Mwandawiro C, Kihara J, Simiyu E, Gitonga CW, Noor AM (2015). Comparing insecticide-treated bed net use to *Plasmodium falciparum* infection among schoolchildren living near Lake Victoria, Kenya. Malar J.

[65] Biran A, Smith L, Lines J, Ensink J, Cameron M (2007). Smoke and malaria: are interventions to reduce exposure to indoor air pollution likely to increase exposure to mosquitoes?. Trans R Soc Trop Med Hyg.

[66] De Meillon B (1930). *Anopheles funestus* (Giles) in smoke-filled native huts. J Med Assoc S Afr.

[67] Symes CB (1930). Anophelines in Kenya. Kenya East Afr Med J Nairobi.

[68] Gibbins EG (1933). The domestic *Anopheles* mosquitoes of Uganda. Ann Trop Med Parasitol.

[69] Po JYT, FitzGerald JM, Carlsten C (2011). Respiratory disease associated with solid biomass fuel exposure in rural women and children: systematic review and meta-analysis. Thorax.

[70] Ferguson HM, Dornhaus A, Beeche A, Borgemeister C, Gottlieb M, Mulla MS (2010). Ecology: a prerequisite for malaria elimination and eradication. PLoS Med.

[71] The malERA Consultative Group on Vector Control (2011). A research agenda for malaria eradication: vector control. PLoS Med..

[72] Killeen GF (2014). Characterizing, controlling and eliminating residual malaria transmission. Malar J.

[73] Amek N, Bayoh MN, Hamel M, Lindblade KA, Gimnig JE, Odhiambo F (2012). Spatial and temporal dynamics of malaria transmission in rural Western Kenya. Parasit Vectors.

